# Healthful Breathing

**Published:** 1887-02

**Authors:** 


### HEALTHFUL BREATHING.

Tight lacing and lazy ways of breathing, says Helen C. Swazy, in *St. Nicholas*, prevent the lungs of the adult woman from getting enough exercise for their own good.

It is well to establish the habit of deep breathing, but, in addition to this, the reserve air which is left in the lungs after an ordinary expiration should be expelled and the lungs thoroughly ventilated at least twice every day. First, then, see to it that the air in the room is as pure and fresh as out-of-door air can make it. Then, with all tight and superfluous clothing removed, lie flat on the back and, with the mouth firmly closed, take a full deep breath. Hold it eight or ten seconds, and then let it out. Take another, and yet another breath in the same way.

After that, take a breath into the lungs as slowly as possible, beginning to fill them up at the lowest extremities, and inhaling gradually until they are filled to their full capacity, when the air should be exhaled in the same slow and steady manner in which it was taken in.

When you have taken this movement again to make sure that the shoulders are in good position, throw yours arm vertically over your head and take another quick, full inspiration, swinging the arms rapidly to the sides close to the body and back again over the head. Swing the arms up and down four times on the same breath and repeat the exercise three or four times.

After this it is a good plan to stand erect with the arms horizontal at the sides and vigorously clasp the hands from that position over the head a few times. When taking such movements in an erect position, always keep the chin two or three inches back of the vertical.

A few such exercises as these for five or ten minutes at night and morning will promote refreshing sleep and give increased vitality.

